# Injury in China: a systematic review of injury surveillance studies conducted in Chinese hospital emergency departments

**DOI:** 10.1186/1471-227X-11-18

**Published:** 2011-10-26

**Authors:** Michael Fitzharris, James Yu, Naomi Hammond, Colman Taylor, Yangfeng Wu, Simon Finfer, John Myburgh

**Affiliations:** 1 Accident Research Centre and Injury Outcomes Research Unit, Monash Injury Research Institute, Monash University, Victoria, Australia; 2 Critical Care and Trauma Division, The George Institute for Global Health, Sydney, Australia; 3 Research and Development, The George Institute for Global Health, Beijing, China; 4 Office of the Director, The George Institute for Global Health, Beijing, China; 5 Peking University Clinical Research Institute, Peking University Health Science Center, Beijing, China; 6 Faculty of Medicine, University of Sydney, Sydney, Australia; 7 Faculty of Medicine, University of NSW, Sydney, Australia; 8 Department of Intensive Care Medicine, St George Hospital, Sydney, Australia

## Abstract

**Background:**

Injuries represent a significant and growing public health concern in China. This *Review *was conducted to document the characteristics of injured patients presenting to the emergency department of Chinese hospitals and to assess of the nature of information collected and reported in published surveillance studies.

**Methods:**

A systematic search of MEDLINE and China Academic Journals supplemented with a hand search of journals was performed. Studies published in the period 1997 to 2007 were included and research published in Chinese was the focus. Search terms included emergency, injury, medical care.

**Results:**

Of the 268 studies identified, 13 were injury surveillance studies set in the emergency department. Nine were collaborative studies of which eight were prospective studies. Of the five single centre studies only one was of a prospective design. Transport, falls and industrial injuries were common mechanisms of injury. Study strengths were large patient sample sizes and for the collaborative studies a large number of participating hospitals. There was however limited use of internationally recognised injury classification and severity coding indices.

**Conclusion:**

Despite the limited number of studies identified, the scope of each highlights the willingness and the capacity to conduct surveillance studies in the emergency department. This *Review *highlights the need for the adoption of standardized injury coding indices in the collection and reporting of patient health data. While high level injury surveillance systems focus on population-based priority setting, this *Review *demonstrates the need to establish an internationally comparable trauma registry that would permit monitoring of the trauma system and would by extension facilitate the optimal care of the injured patient through the development of informed quality assurance programs and the implementation of evidence-based health policy.

## Background

The magnitude of injury-related mortality and morbidity in China was highlighted by Wang and colleagues in the *Health System Reform in China Series *featured in *The Lancet*[1]. Wang et al. reported that 10% of all deaths and 30% of all potentially productive life years lost (PPYLL) were due to injury related causes. In numeric terms, this equates to the loss of approximately 850,000 lives per annum, with two-thirds of those killed being less than 45 years of age [1]. Traffic-related injury (32.3%), drowning (13.4%), falls (9.7%) and poisoning (4.5%) were the leading causes of unintentional injury deaths, while suicide was the leading cause of intentional injury and the second leading cause of injury deaths (23%) overall. Injuries represent the leading cause of death for persons under 40 years of age [2,3].

With close to 23 million disability-adjusted life years (DALYs) lost per annum (11.5% of all-cause DALYs), unintentional injuries represent a significant source of morbidity. Road traffic crashes account for one-third of these DALYs, followed by 'other unspecified causes' (29%), falls (17%), drowning (15%) and poisonings (6%)[4]. An estimated 200 million persons are injured each year, with approximately one-third (62 million) requiring emergency care or hospitalisation [5]. The consumption of health resources as a consequence of injury is significant. Direct medical costs have been estimated to be as high as CNY 64.1 billion RMB (USD$9.3 billion) per annum, with costs related to delay and absence from work being approximately CNY 6 billion (USD$0.8 billion)[5], equivalent to 1.92% of GDP (2007) [6].

Within this context of high injury rates and perceived limited available epidemiological data, commentators have identified the need for the establishment of population based injury surveillance systems to guide public health programs [3,7,8]. A number of fatality reporting systems and data sources do however exist, these being the *National Statistics Yearbook*, the *Transportation Statistics Yearbook*, and the *Health Statistic Yearbook*, the latter which reports mortality statistics for select causes of injury. While cause-of-death data leads to an understanding of changing disease patterns and permits population health policy planning, hospital-based injury surveillance systems and trauma registries facilitate prevention efforts as well as forming the basis of hospital quality assurance programs [9]. It has been noted that to date such systems have been limited in their scope within China [3,10,11].

Given the high incidence of injury in China and the calls for the establishment broad based injury surveillance programs, it was considered timely to document the extent to which injury surveillance studies have been conducted. Whilst also documenting the incidence and causes of injury for a wider audience, this *Review *aims to document existing research strengths as well as areas of surveillance systems research that require strengthening. Of particular interest was the extent to which the reporting of patient injury data is consistent with commonly accepted global reporting guidelines, and whether there is a need for broad-based injury surveillance and/or trauma registry systems to be implemented. In conducting this *Review*, there were two specific objectives:

1. To describe the characteristics of persons presenting to an emergency department following injury and the associated mechanisms of injury, and

2. To document that type of patient and injury information commonly reported, and following this, determine the extent to which this reporting is consistent with commonly accepted global guidelines.

## Methods

### Protocol and registration

This systematic *Review *has not been registered. The research objectives, analysis methods and inclusion criteria are fully specified here.

### Study eligibility criteria

Retrospective and prospective studies of injured persons presenting to an emergency department in China were the focus of this *Review*. Studies that included all-cause injury presentations published from 1997 to 2007 in the Chinese language were included. Studies that focused exclusively on traffic crashes, age cohort subsets or specific injuries were excluded from the *Review*.

### Information sources

Studies were identified using electronic databases, a hand-search of the Tables of contents pages of general and specialist medical Chinese language journals (Table 1), and by scanning reference lists of identified articles. The initial search strategy included both Chinese and English language articles within the limits specified above, with 'Medline' and 'China Academic Journals Full-Text Database' used. The last search was performed in 11 July 2009.

**Table 1:** List of Journal outlets searched by hand

Chinese Journal of Epidemiology
Chinese Journal of Critical Care;

Chinese Journal of Traumatology

Chinese Journal of Industrial Hygiene and Occupational Medicine

Chinese Journal of Disease Control and Prevention

Chinese Critical Care Medicine

Journal of the Fourth Military Medical University

Journal of Sichuan University (Medical Science Edition)

Chinese Journal of Hospital Administration

Chinese Journal of Geriatrics

Chinese Hospital Management

Chinese Journal of Neurosurgical Disease

Journal of Tongji University (Medical Science)

Journal of Traumatic Surgery

Shanghai Journal of Preventative Medicine

Journal of Xinxiang Medical College

Orthopaedic Journal of China

Chinese Journal of Emergency Medicine

### Search strategy

For the computerised searches the following search terms were used: 'China'; 'emergency medical services'; emergencies; emergency; ambulances; air ambulance(s); 'emergency service, hospital'; 'emergency department'; 'pre-hospital care'; 'wounds and injuries'; accident(s). Multiple searches (4) were conducted and duplicate articles were identified and eliminated. The same search terms and strategy was used in both electronic databases (Table 2 appendix).

**Table 2:** Appendix - SEARCH STRATEGY - MEDLINE (OVID)

Search 1	
01	China.mp or China/

02	Emergency Medical Services or emergency medical services.mp

03	Emergencies or emergencies.mp

04	2 or 3

05	1 and 4

06	limit 5 to humans

07	limit 6 to Chinese

**Search 2**	

01	China.mp or China/

02	Ambulances or Air Ambulances or ambulance.mp or pre-hospital care.mp

03	1 and 2

04	limit 3 to humans

05	limit 4 to Chinese

**Search 3**	

01	China.mp or China/

02	Emergency Service, Hospital or emergency department.mp.

03	1 and 2

04	limit 3 to humans

05	limit 4 to Chinese

**Search 4**	

01	China.mp or China/

02	injury.mp. or "Wounds and Injuries"/

03	accident.mp. or Accidents/

04	2 or 3

05	1 and 4

06	limit 5 to humans

07	limit 6 to Chinese

### Study selection and classification of the identified research

**Figure 1:**
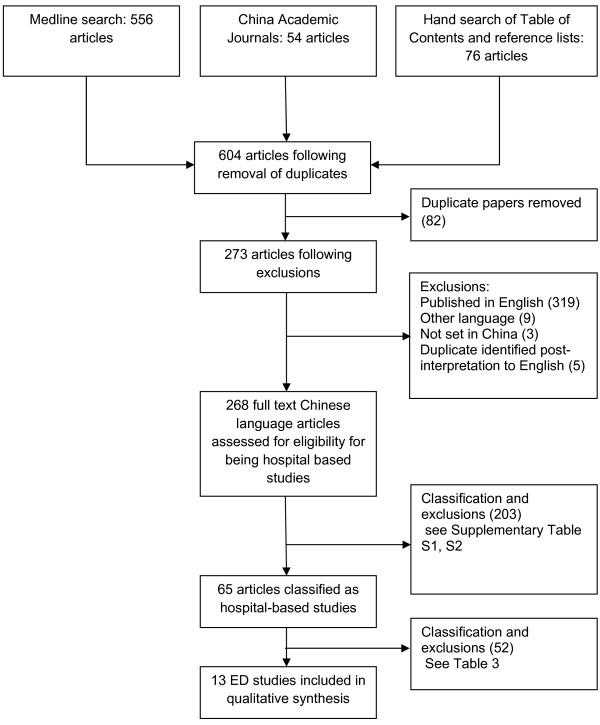
**Number of identified, screened, eligible and included original research articles in the review process**.

One *Review *author (MF) conducted the searches and with the assistance of *Review *Author JY classified each study according to its principal focus. A classification scheme was developed (refer Additional file 1 Table S1) with the number of published papers in each category noted (refer Additional file 2 Table S2). Figure 1 presents the identification, screening, eligibility assessment and included studies in accordance with the PRISMA specification [12].

### Data items of interest

In seeking to fulfill the second aim of this *Review*, the patient characteristics, injury severity and outcome indicator data fields of interest were specified a-priori. As the primary interest was in determining the comparability of reported data with internationally recognized best practice, the fields of interest were motivated by reference to the *Utstein Style 'R*ecommendations for uniform reporting of data following major trauma' [13] and its subsequent revision [14], the *Australia and New Zealand National Trauma Registry *[15] and the American College of Surgeons *National Trauma Databank *[16] and *Resources of Optimal Care of the Injured Patient*[9]. The identified data fields are presented in Table 3 and each study included in the *Review *is compared across these data fields. In this *Review *it was considered too complex to include all data points from the above four reference documents; rather the items selected were done so on the basis of being the minimum key parameters required for comparisons across international studies. Particular attention was paid to whether studies reported the Abbreviated Injury Scale [17], the Injury Severity Score (ISS) [18], ICD codes [19], the Glasgow Coma Score [20], the Revised Trauma Score [21] and the Trauma Injury Severity Score (TRISS) [22].

**Table 3:** A-priori identified patient characteristic, injury severity and outcome indicator data fields of interest

*Study design*	*Factors related to the circumstances of injury*
Design (prospective, retrospective)	Injury mechanism (transport - detail, fall, assault, poisons, burns etc...)

Setting (location)	Location of injury (road, industrial, home)

Number of hospital ED's in study	***Injury coding and clinical indicators***

Data collection source/Survey tool (registry, survey)	Abbreviated Injury Score (AIS) [17]

Date of study	Injury Severity Score (ISS) [18]

Sample size	International Statistical Classification of Diseases and Health Related Problems (ICD) coding [19]

**Patient factors**	Glasgow Coma Score (GCS) [20]

Age	Revised Trauma Score (RTS) [21]

Gender	Trauma Injury Severity Score (TRISS) [22]

Occupation	**Outcomes**

***System factors***	Admission to ICU

Pre-hospital care/mode of arrival	Mortality rate

Presentation/admission to ED (*inclusion criteria of Review)*	Length of stay

	Financial costs

### Data collection process

Using the a-priori identified *data items *of interest data was entered into a MS Excel Spreadsheet for the 13 relevant studies. One author (MF) performed the initial data extraction which was verified by Author JY. *Review *author YW further resolved questions of interpretation from Chinese to English in the source articles.

## Results

Thirteen research papers were identified that met the *Review *inclusion criteria [23-35]. The three search strategies identified 273 scientific papers, of which 143 were identified from Medline, 76 via the manual hand search and 54 from Chinese Academic Journals database. There were 268 unique papers following exclusion of five identified duplicate papers with 65 being hospital-based studies; of these, 13 were injury surveillance studies based in the emergency department (Table 4 Figure 1).

**Table 4:** Article sub-types for hospital-based injury studies

Focus of article	Number
Emergency Department injury surveillance	13

Inpatient injury surveillance	3

Management of injury, practices	8

Injury secondary to disease	0

Body region specific injury	

Traumatic brain injury	4

Face	4

Thorco-abdominal	3

Extremities	2

Multi-trauma	1

Age group specific	

Paediatrics	1

Child	11

Older adults	6

Retrospective prediction mortality model	6

Cause specific	

Poisoning	3

Total	65

### Description of the identified studies: patient characteristics and injury mechanisms

The 13 emergency department injury surveillance studies (nine prospective; four retrospective) were grouped into four categories: 1. the '25 emergency department's studies'; 2. Prospective studies using the National Injury Surveillance System (NISS) Reporting Card; 3. Collaborative studies, and 4. Single centre studies. Table 5 details the key aspects of each study and highlights the type of patient information collected. A brief description of each study is presented below both to provide the context for a discussion on the type of patient data collected and to fulfil Aim 1 of increasing the accessibility of Chinese injury surveillance research; in the main, the data discussed below is not presented in the Tables.

**Table 5:** Summary of key study characteristics

	25 ED study	25 ED study	25 ED study	NISS RC study	NISS RC study	NISS RC study	NISS RC study	Collab. Study	Collab study	Single centre	Single centre	Single centre	Single centre	Single centre
	**Chen et al **[23]	**Li et al **[24] **City**	**Li et al **[24] **Rural**	**Zhang & Zhan **[25]	**Zhou et al **[26]	**Xu et al **[27]	**Li et al **[28]	**Li & Wang **[29]	**Li et al **[30]	**Li et al **[31]	**Qu et al **[32]	**Zhou et al **[33]	**Yang et al **[34]	**Wen et al **[35]

**Design**	Prosp.	Prosp.	Prosp.	Prosp.	Prosp.	Prosp.	Prosp.	Retro.	Prosp.	Prosp.	Retro.	Retro.	Retro.	Retro. (pre/post)

**Setting/location**	Multiple Provinces	Multiple Provinces	Multiple Provinces	Qing-dao, Shangdong	Henan	Guangdong	Gaocheng, He Bei	Guang-dong	Shantou, Guang-dong	Shantou, Guang-dong	Hangzhou, Zhejiang	Guangzhou, Guangdong	Guangzhou, Guangdong	Chonqing

**Number Hospital**	25	14	11	6	3	10	26	332	2	1	1	1	1	1

**Sample**	25,019	19,906	5113	1882	6948	42,567	7065	1,093,233	2611	11,472	13,008	10,654	5346	8271

**Survey tool**	Uniform survey	see [23]	see [23]	National CDC RC	National CDC RC	National CDC RC	National CDC RC	Report form	Uniform survey	Uniform survey	Registry log	Not stated	Registry log	Uniform survey

**Date of study**	July 01 Oct 01 Jan 02 April 02	see [23]	see [23]	2004	2004	2004	Dec 03 to Nov 04	1997-2001	Nov 99 - Nov 00	2000-2002	1998-2002	2000-2005	Aug 03-Aug 05	1996-2004

**Age** **(years)**	0-14: 12% 15-34: 50% 35-59: 31% 60+: 7%	see [23]	see [23]	< 21: 22% 21-59: 71% > 60: 4%	0-14: 6%; 15-44: 66% 45-64: 14% 65+: 4%	12-24: 31% 25-34: 29%	0-4: 2% 5-14: 8% 15-19: 14% 20-24: 13% 25-44: 39% 45-64: 19% 65+: 5%	-	20-35: 47% no specific data	0-19: 25% 20-39: 55% 40-59: 15% 60+: 5%	-	0-10: 2% 11-20: 7% 21-30: 33% 31-40: 25% 41-50: 20% 51+: 11%	0-15: 10.3% 15-20: 14% 21-30: 32% 31-40: 20% 41-50: 11% 51-60: 5% 60+: 8%	*Range*: Pr: 14-86 Po:16-79 *Mean*: Pr: 32 Po: 34

**Sex ratio (M:F)**	2:1	2:1	2:1	3.1:1	2.3:1	2.5:1	2.5:1	-	2.5:1	2.6:1	-	2.4:1	1.9:1	Pr:1.6:1 Po: 1.9:1

**Occupation**	-	Yes	Yes	Yes	Yes	-	-	-	-	-	-	-	-	-

**Arrived by EMS**	14.4%	14.4%	14.4%	29.4%	-	-	-	-	-	-	-	-	-	-

**Mechanism**	Yes	Yes	Yes	Yes	Yes	Yes	Yes	Yes	Yes	Yes	Yes	Yes	Yes	Yes

**Location of injury**	-	-	-	Yes	Yes	Yes	-	-	-	-	-	-	-	-

**ISS/AIS severity**	-	-	-	-	-	-	-	-	-	-	-	ISS category	Deaths only	AIS ≥ 3 ISS > 15

**ICD**	-	-	-	-	-	-	-	-	-	-	-	-	-	-

**GCS**	-	-	-	-			-	-	-	-	-	-	-	-

**Injury description**	-	-	-	-	Yes	Yes	-	-	-	-	-	-	-	-

**RTS**	-	-	-	-	-	-	-	-	-	-	-	-	-	-

**TRISS**	-	-	-	-	-	-	-	-	-	-	-	-	-	-

**ICU admission**	-	-	-	-	-	-	-	-	-	-	-	-	-	-

**Mortality rate**	0.5% P: 1.1% AI: 0.5%	0.5%	1.29%	-	-	-	0.8%	1.6%	4% (within 3 months)	-	1.3%	Pre-hosp: 3.4%; ED:NR	0.3%	Pr: 7.6% Po: 3.2%

**Length of stay**	-	-	-	-	-	-	-	16 days	-	-	-	-	-	-

**Financial cost**	-	-	-	-	-	-	-	Yes	-	-	-	-	-	-

Abbreviations: RC: Reporting Card; Collab: Collaborative study; (-) indicates data not reported; Prosp: Prospective; Retro: Retrospective; Unint: Unintentional; NR: Not reported; Pr: Pre-phase of study; Po: Post-phase of study; ED: Emergency Department; P: Poisoning; AI: Acute Injury

### The '25 emergency departments' study

The '25 emergency departments' study aimed to determine the type of patients attending hospital due to injury, to report the mode of transportation to hospital, and to document mortality outcomes. This study was reported in two papers [23,24].

As a way of examining the feasibility of establishing a hospital-based injury surveillance system, Chen et al implemented a 'uniform survey' proforma to prospectively collect patient data in 25 hospitals [23]. The 'census' dates were the 1^st ^- 15^th ^in each of July 2001, October 2001, January 2002 and April 2002 (Table 5). In the 60 day period, 143,274 patients presented to ED of which 25,019 (17.4%) patients presented due to injury. Of these, 91.4% were described as having sustained 'acute injury' and 8.6% as 'poisoning'. The overall injury mortality rate was 0.5% although mortality was higher for poisonings (1.1%) than for acute injury (0.4%) patients. The leading cause of injury was reported as 'mechanical injury' in the industrial and farming context (32.7%) followed by traffic crashes (26.9%, 6147). Traffic crashes accounted for nearly 47% of deaths. The male to female ratio was 2:1 for age groups under 60, above which the ratio was 1.07:1. Only 14.4% were transported to the emergency department by emergency vehicle with the remainder described as 'other means' or 'private'.

Using the same data, Li et al reported that injury-related admissions were higher in the 11 rural hospitals (29%) compared to the 14 city hospitals (19%), as was the mortality rate (rural: 1.29%; city 0.27%)[24]. Transport accounted for 35% of injuries in rural hospitals followed by industrial machine type injuries (18.15%), whereas the reverse was true for city hospitals (industrial machine type injuries: 33%; transport: 21.8%). The study collected and reported upon employment status, one of only three in this *Review *to do so (Table 6). Transportation workers (22%, 74% male) and students (12.7%, 60% male) were the leading occupations in the city cohort, while in the rural hospitals farmers (37%, 72% male), students (14%, 74% male) and transport workers (9%, 87% male) were the leading occupations. Mortality was the only clinical outcome variable reported in the study.

**Table 6:** Patient-focussed clinical parameters reported in the Reviews

Parameter	% of studies (number of references)	Comments and detail
***Patient Factors***		

Age distribution (years)	84% (11 of 13)	• No uniformity of categories [23-26,28,31,33,34], failure to report of full age range [27,30,35]

Sex distribution (M:F)	84% (11 of 13)	• Reported number and % male & females [23-28,30,31,33-35]

Occupation	23% (3 of 13)	• Transportation worker, student, farmer, technical worker, service workers [24] • Worker, farmer, fisher [25] • Worker, student, farmer/forester/fisher [33]

***System Factors***		

Pre-hospital care	23% (3 of 13)	• EMS or other [23,24]; medical aid [25]

***Factors related to circumstances of injury***		

Mechanism	100%	• 'Super-categories' (i.e., unintentional, transport) • Non-uniform use of transport/traffic

Location	23% (3 of 13)	• Industrial, road/street, home, school [25] • Road/street; family, operational site [26] • Operational site; road street [27]

**Injury Coding and clinical indicators**		

Abbreviated Injury Score severity [35]	7.7% (1 of 13)	• For isolated trauma AIS ≥ 3 [35]

Injury Severity Score [17]	23% (3 of 13)	• For those admitted to ED, ISS: < 16; 16-24 [33] • For deaths only [34] • For multi-trauma ISS ≥ 15 [35]

ICD [18]	None	

'Other injury' description	15% (2 of 13)	• Superficial; open; fracture (not by region) [26] • Superficial; open; fracture (not by region) [27]

GCS [16]	*None*	

RTS [19]	*None*	

TRISS [20]	*None*	

**Outcomes**		

Mortality	69% (9 of 13)	• Overall mortality plus traffic separately [29] • % within 3-months [30] • Pre-hospital mortality only [33] • Based on registry log only [34] • Patient mortality in ED [23,24,28,32,35]

Admission to ICU	*None*	

Length of stay	7.7% (1 of 13)	• Mean given [28]

Costs	7.7% (1 of 13)	• Costs per stay [28]

Reference to the a-priori established indicators of interest (Table 3 Table 5) highlights that no injury coding or clinical indicators were collected and reported in this study program. Despite this, the study was successful in establishing a comprehensive network that could serve as the basis for more detailed injury surveillance or integrated trauma registry systems.

### Prospective Studies using the National Injury Surveillance System Reporting Card

Four studies [25-28] that utilised the Chinese-Centre of Disease Control (C-CDC) NISS Reporting Card [36] were identified (Table 5). The Reporting Card commenced widespread use in late 2005 as the basis of NISS, later than the publishing date of these studies. Each study collected data prospectively at three [26], six [25], 10 [27] and 26 [28] hospitals for a period of 12-months, reflecting the expansion of NISS. The Reporting Card collects basic patient, mechanism, and outcomes; however the studies were mixed in the reporting of these aspects (Table 5). For instance, only one of the four reported mortality [28], one reported arrival by EMS [25], two noted occupation [25,26] and two provided a simple description of injuries sustained but without reference to body region [26,27]. The reported age categories also differed, with Li et al. [28] providing the most comprehensive. Notable aspects of each study are described below with detail provided in Tables 5, 6 and 7.

**Table 7:** Leading causes of injury in the Reviewed studies, with WHO Global Burden of Disease incident cases

'Review' Study	Author	1	2	3	4	5	6	7
**'25 Emergency Departments' study**	Chen et al [23]	Machine (29.9%)	Transport (24.6%)	Assault (17.8%)	Falls (16.7%)	Poisons (8.6%)		

	Li et al [24]: City	Machine (32.9%)	Transport (21.8%)	Falls (17.9%)	Assault (13.5%)	Poisons (4.7%)		
	
	Li et al [24]: Rural	Transport (35.3%)	Machine (18.2%)	Falls (12%)	Poisons (7.9%)	Assault (7.1%)		

**NISS Reporting Card studies**	Zhang & Zhan [25]	Blunt (28.6%)	Traffic (26.8%)	Falls (16.5%)	Cutting/piercing (8.9%)			
	
	Zhou et al [26]	Blunt (28.1%)	Transport (23.8%)	Falls (18.3%)	Cutting/piercing (10.3%)	Poison (6.5%)		
	
	Xu et al [27]	Blunt (25.8%)	Falls (25.8%)	Traffic (16.8%)	Cutting/piercing (15.6%)	Other (6.7%)		
	
	Li et al [28]	Transport (36%)	Blunt (25%)	Falls (17%)	Cutting/piercing (9%)	Poisons (6.4%)		

**Collaborative studies**	Li & Wang [29]	MVA (36.1%)	Falls (15.3%)	Industrial (11.9%)	Assault (16.8%)	Other (25.1%)		
	
	Li et al [30]	Unintentional (80.8%)	Assault (15.6%)	Suicide (3.6%)				

**Single-centre studies**	Li et al [31]	Traffic (38.4%)	Suicide (15.9%)	Assault (12.8%)	Falls (12.2%)	Cutting/piercing (6.2%)		
	
	Qu et al [32]	Transport (77.8%)	Machine (9.6%)	Falls (8.5%)	Cutting/piercing (1.2%)			
	
	Zhou et al [33]	Transport (39.2%)	Assault (32.7%)	Machine 12.9%)	Burns (2.6%)			
	
	Yang et al [34]	Cutting/piercing (41.4%)	Falls (27.6%)	Transport (23%)	Machine (3.7%)	Burns (3.1%)		
	
	Wen et al [35]	Traffic (53.4%)	Falls (20.9%)	Cutting/piercing (9.7%)	Assault (10.3%)	Machine (4.5%)	Firearms (0.21%)	

**Comparator studies**								

**Global Burden Disease (WHO Regions) **[46]	The Americas	Violence (35.9%)	Other unintentional^‡ ^(27.8%)	Falls (19.8%)	Road traffic crashes (13.3%)	Poisoning (2%)	Fires (1%)	Drowning (0.1%)
	
	Europe	Falls (36.4%)	Other unintentional^‡ ^(33.9%)	Road traffic crashes (12.4%)	Violence (11.1%)	Fires (3.7%)	Poisoning (2.3%)	Drowning (0.1%)
	
	South-East Asia (incl. India)	Other unintentional ^‡ ^(40.4%)	Falls (28.4%)	Road traffic crashes (16.9%)	Fires (8.3%)	Violence (4.3%)	Poisoning (1.6%)	Drowning (0.1%)

	Western Pacific (incl China, Aust)	Other unintentional ^‡ ^(34.9%)	Road traffic crashes (19.2%)	Violence (4.7%)	Poisoning (2.5%)	Fires (1.8%)	Drowning (0.1%)	

**Country specific**	Australia [47] (hospitalisation)	Falls (36%)	Transportation (14%)	Intentional self-harm (6%)	Assault (6%)	Poisoning, pharmaceuticals (2%)	Fires, burns and scalds (1%)	
	
	United States [48] (hospitalisation)	Motor vehicle, cyclist, pedestrian (39.8%)	Falls (30.2%)	Struck by, against (6.7%)	Transport, other (5.3%)	Firearm (5.3%)	Cut/pierce (4.9%)	Fire/burn + flame (2%)

**EU-region**	EU-27 [49] (fatal)	Self-inflicted (23%)	Road Traffic (20%)	Falls (17%)	Poisoning (5%)	Drowning (3%)	Interpersonal violence (2%)	Fires (2%)
	
	EU-27 [49], hospital admissions	Home, leisure (63%)	Road Traffic (14%)	Sports (8%)	Suicide, self-harm (6%)	Homicide/assault (4%)	Workplace (4%)	School (1%)

‡Balance of external causes in ICD10 V01-X59, Y40-Y86, Y88, Y89

Zhang and Zhan [25] reported the characteristics of 1882 patients in six hospitals in the Huangdao district of Qing-dao city. 'Blunt instrument injury' (28.6%) and traffic related injuries (26.8%) were the two dominant injury mechanisms. The use of broad age categories used resulted in 71% falling into the single 21-59 year age category, with 22% under 21 years and 3.6% above 60 years. The male to female ratio was 3:1, the highest of any of the 'collaborative studies' reported here. Occupation was reported using the terms generic 'worker' (53%), farmer/fisherman (14.4%) and students (11%). Over one-third of patients were injured in an industrial environment followed by the road, the home and at school. Only 29.4% received pre-hospital medical aid, this being the only key a-priori clinical system indicator reported.

A similar pattern of injury mechanism - with the addition of poisons being reported, can be seen in the study that involved 6948 patients presenting to two Level 3 hospitals (elite) and one Level 1 hospital in the Henan Province, reported by Zhou, Zhang and Li [26]. The age group structures differed from all other papers in this *Review*, with 0-14 years (6%), 15-44 years (66%), 45-64 years (13.9%) and 65+ years (6.5%) being used. The study was one of only two in the *Review *to report injury details however these were reported as superficial wounds (28.7%), open wounds (25%) and fractures (16.3%) without reference to body region. None of the key clinical indicators of interest were reported. This study is important as the stated aim was to set up a surveillance system to guide injury prevention policy priority setting. The authors concluded that traffic management, safety programs focussed on the young, and preventative programs targeting older adults' falls in the home were critical.

In the largest study of the Reporting Card series, Xu et al [27] reported on 42 657 patients at 10 hospitals including two Level 3 (elite) hospitals and one Level 1 hospital in each of two cities, as well as one county level hospital and one village level hospital in Guangdong Province. Blunt instrument wound was the most common mechanism (29.8%), followed by falls (25.8%), and then traffic crashes (16.8%). Limited age data was reported, with only two categories noted: 12-24 years: (31.4%) and 25-34 years (29.3%) (Table 5). Injury location specified as operational (industrial) site (41%) and roads (31%), these being different descriptors to other studies in the *Review*. The same injury descriptions as Zhou et al [26] with superficial wounds (35.9%), open wounds (33.8%) and fractures (10.7%) were used. None of the key injury severity and outcome indicators of interest were noted. Despite this limitation, the study is important as the stated intent was to highlight the importance of surveillance systems as the basis for injury control strategies.

In the fourth of the Reporting Card studies, Li et al [28] reported on 7065 patients who presented to one of 26 hospitals in Gaocheng due to injury. Similar mechanism categories as the other studies were used, with transport (36%) and blunt instrument (25%) being the leading causes of injury. The reporting of age in this study was the most comprehensive all papers in the *Review*, particularly for those under 25 years of age. This was the only one of the four 'reporting card' studies to report mortality, with the mortality rate being 0.86%. No other key indicators of injury severity or patient outcomes were noted.

### Collaborative studies

Two studies were identified as being 'collaborative studies', one being a retrospective study of patients admitted to 332 hospitals in Guangdong over a 5 year period [29] and the other a prospective study at two hospitals in Shantou over a 1-year period [30] (Table 5).

Li and Wang's 1997-2001 retrospective study [29] is the largest reported in this *Review*, with nearly 1.1 million patients admitted to an emergency department due to injury. Data was collated from *Reporting Forms *sent by the hospitals to a central health authority. As with all of the studies, injury mechanism was documented using standard categories, these being motor vehicle crashes (36%), unintentional falls (15.3%), industrial accidents (11.9%), and assault (16.8%) (Table 7). Despite some similarity in reporting categories, the ICD system was not used. The overall mortality rate was 1.6% with 56% being traffic-related deaths. This was the only study in the *Review *to report mean length of stay (16 days) as well as cost of treatment. The mean cost for treatment was CNY 5442 (USD$790) equating to approximately CNY 5.9 billion (USD$0.86bn) for the presenting patients across the 5 years at the participating hospitals. The study did not report age, gender, occupation, or location of injury, nor were any of the clinical severity indicators reported.

Li et al [30] provided details of 2611 patients presenting to two hospitals in Shantou over the period of one year (Nov 1999 - Nov 2000). The authors used a survey designed specifically for the study, although as presented the data was limited to a broad description of injury mechanism (i.e., [un]intentional) and a single limited age category (20-35 years: 47%). Mechanism of injury was ill-defined, with approximately 81% of patients presenting to the ED due to unspecified 'unintentional injuries', 15.6% due to assault, and 3.6% suicide. The mortality rate, noted as being within 3-months of injury, was 4%. No other indices of severity, length of stay or injury information were presented.

### Single centre studies

Five single centre studies were identified, with the patient sample size ranging from 5436 [34] to 13 008 patients [32] with all being three or more years in duration (Table 5). Only one study was prospective in design [31], with four being retrospective reviews. All reported mechanism of injury although categories varied (Table 7), all but one [32] reported age data, and one study failed to note the sex distribution of the sample [32]. With respect to the key outcome indicators, none of the studies reported length of stay, head injury or GCS, RTS, TRISS, financial costs, or pre-hospital care; in addition, none reported patient occupation, or location. Transport was the leading cause of injury in all but one study where cutting/piercing (41%) was the leading injury mechanism [34] (Table 7).

Li et al [31] set out to examine violence as an injury mechanism, and in doing so collected data in a prospective manner on 11 472 patients in a 3 year period using a purpose designed survey. Mechanism of injury, age, and the sex distribution was described (M:F 2.6:1), however there was no data concerning key injury severity and outcome indicators. The leading mechanisms were traffic (38.4%), suicide (15.9%) and assault (12.8%). Young adults (20-39) accounted for 56% of all patients. Four age categories were used, permitting only a limited understanding of injuries experienced by young children and older adults.

The retrospective study of 13 008 patients at one hospital in Hangzhou reported by Qu et al [32] used the emergency department registry log as the basis for analysis, and reported only mechanism and mortality statistics (1.3%). In contrast to all other studies in this *Review*, three-quarters of the patients presented due to injury sustained in a transport-related crash, followed by machinery (9.6%) and falls (8.5%). Aside from these details noted above, the study presented limited patient characteristics, injury event, clinical indices and outcome variables (Table 5).

In a 5 year study published in 2006 [33], Zhou et al reported on the characteristics of 10 654 patients presenting the emergency department. Of these, 361 died (3.4%) prior to admission to the ED and 568 (5.3%) either refused treatment or were transferred to other hospitals. This was the only study to report pre-hospital deaths however mortality of those 'admitted' to the ED was not reported. The age distribution was divided into 10-year intervals, with those aged 20-30 years accounting for 33% of all presentations although the age distribution was capped at 51+ years, the lowest of any of the studies here (Table 5). The ISS was calculated for the 9725 patients admitted and treated in the ED (ISS < 15: 62%; 16-24:22%; > 24: 16%), one of only three studies in this *Review *to do so (Table 6).

Yang et al's [34] 2-year study of 5346 patients presenting to one hospital in Guangzhou following injury used a registry log as the data source. Mechanism was reported and while the descriptive categories were similar, the injury cause profile differed from all other studies with cutting/piercing being the most common injury mechanism (41%) (Table 7). No information concerning patient occupation or location of injury was presented. The age categories included children and youth combined (0-15), then used deciles with the upper category being 61+ years; none of the studies in this *Review *categorised older adults in detail with age being capped in the mid-60's or being 60+ years. Reliance on the initial registry log meant that only nine deaths were recorded, with the ISS being recorded only for these patients (0.3%), presumably due to later examination or autopsy, although this was unclear.

The patient series presented by Wen et al [35] was a pre-post comparison on the establishment of a dedicated emergency trauma department. The 'pre-period' was 1 January 1996 to 31 December 1997 with patients being assigned to a surgical department for care (i.e., usual care). The 'post-period' was 1 January 1998 to 1 January 2004 (75% of patients), with patients treated within a dedicated trauma department. The study captured 8271 patients, of which 53.3% (4416) were injured in road traffic crashes (Table 5, 7). Age was reported as a mean and a range, while gender, mortality and injury mechanism were also reported. The study reported AIS for patients with an isolated injury (the only study to use AIS in the *Review*) and ISS for multi-trauma patients. For patients in the 'pre' trauma service period 74% (1269 of 1715) had an AIS ≥ 3 injury compared to 77% (3998 of 5192) AIS ≥ 3 injuries in the 'post' period. For the multi-trauma patients, 69% (220 of 318) of patients in the pre-period had an ISS > 15 in contrast to 86% (902 of 1046) of those in the 'post' period. The establishment of the trauma service resulted in a significant reduction in a range of key process and outcome indicators (Table 8). This study is important as it provides evidence that the formation of a dedicated trauma service provides superior care on these performance metrics. The ability to report these findings clearly demonstrates the value and importance of collection and analysis of registry data. In this context it is worth commenting that the purpose of this study was to evaluate trauma system change rather than the surveillance nature of the other studies in this *Review*, and hence the greater emphasis being placed on the collection of treatment processes and clinical outcomes than in the other studies reported in this *Review*.

**Table 8:** Reductions associated with the establishment of a dedicated trauma service compared to allocation to surgical departments, derived from Wen et al [35], and 'post' trauma department benchmark metrics (in parenthesis)

	Isolated injury		Multiple injuries	
**Index**	**Mild, AIS < 3**	**Severe, AIS ≥ 3**	**Mild, ISS < 15**	**Severe, ISS > 15**

Time from admission to diagnosis (min)	52% (34)	59.7% (31)	57% (36)	58% (36)

Time from admission to operation (min)	63% (41)	71% (37)	64% (44)	72% (37)

Length of stay (days)	48% (7)	26% (14)	48% (8)	28.6% (15)

Mortality (%)	No deaths (0)	63% (3.4)	66% (2.8)	52% (6.3)

Complication rate (%)	14% (1.3)	30% (8.5)	23% (15.3)	36% (23.7%)

### Analysis of collected patient-focussed clinical parameters

The second aim of the present *Review *was to determine the type and breadth of patient-focussed data collected in the studies. As noted in the *Method*, a minimum set of data items was specified a-priori as key indicators in the assessment of the identified studies (Table 3). Further to the discussion of each study above, Table 6 shows the number of studies that reported key patient demographic, injury mechanism and location, and severity indices. While all studies reported the mechanism of injury, high-level and mixed category descriptors were used with none using ICD-10 external cause coding (Table 7). Categories such as 'transport', 'traffic', 'unintentional injury' provide only a limited understanding of the mechanism of injury and certainly the use of precise mechanism descriptions - such as pedestrian, motorcyclist, car occupant, as recommended by a range of guidelines are required to permit comparisons between studies to be made and for building a comprehensive national injury profile.

Similarly, while most studies reported the age distribution of their sample there was a lack of uniformity in the age categories used; this was described fully in the text above. There is a need for researchers to adopt the Utstein type age categories [13,14] in order to fully understand injury risk across the age spectrum in China. Two studies failed to report the patient sex, both of these being retrospective studies; these same studies reported patient age in a limited manner. Mortality was the most commonly reported severity index (69%, 9 of 13 studies), however only one study reported pre-hospital mortality. There was little use of standard severity indices. Two studies provided an estimate of superficial, open wounds and fractures but did not differentiate body region, despite the terms 'superficial', 'open' and 'fractures' being used in the ICD. Three studies utilised the AIS-ISS system [33-35] although did so in a limited manner. Only one study reported financial cost data with the same study reporting patient length of stay, these being two inter-related outcome variables. None of the studies in the *Review *reported GCS [20], RTS [21], TRISS [22], ICD codes [19] or admission to ICU.

## Discussion

Set amid growing calls for the establishment of injury surveillance systems in China, we conducted a review of injury surveillance research conducted the emergency departments published locally. The systematic search identified 268 research papers with an injury and medical care focus published in the period 1997 to 2007 published in Chinese; of these 13 were broad-based injury surveillance studies set in hospital emergency departments. While commentators have pointed to the need for the conduct of injury surveillance studies, it is clear that there is an established body of research that has been conducted in the field, some of which involved multiple hospitals and a large number of patients. In addition, there is also a broader body of emergency department research focussed on specific mechanisms of injury (e.g., motor vehicle), age group, or types of injury sustained not reported here.

### Strengths and limitations of the reviewed studies

The studies reviewed have a number of strengths with eight of the thirteen published papers - or 8 of 12 unique studies - being collaborative studies. Seven of the 12 studies reported data collected prospectively, including all but one of the collaborative studies. The co-ordination involved in these large scale studies is noteworthy with data from a large numbers of patients collected over extended time periods.

The reporting of clinical indicators in the collaborative studies was however limited. The six single centre studies provided little additional patient information than the collaborative studies, they ranged from 5 436 to 13 008 patients and were conducted for periods of up to 6 years. In contrast to the collaborative studies, four of the five single centre studies were retrospective in nature. Also in contrast to the collaborative studies, the ISS was reported in three of the five single centre studies; however other key indices such as ICU admission, ICD coding [19], costs and details of injuries by body region were not reported. The pre-/post-trauma service study reported by Wen and colleagues [35] highlighted impressive reductions in key patient outcomes such as length of stay, mortality, complication rates and temporal factors related to care upon establishment of a dedicated trauma service, similar to findings reported previously in the US [37-42].

In all of the studies reviewed, the depth of patient injury data with respect to internationally accepted injury and trauma scoring systems was limited. Only three studies reported the ISS [18], one reported using the AIS for specific injury coding [17], and none used the ICD system to code external cause of injury, type of injury or procedures performed [19]. Furthermore, none of the studies reported the GCS [20], the RTS [21] or the TRISS [22]. The use of standardised and internationally recognised trauma severity metrics is an integral element of health system performance monitoring [9,21,43] and the application of these metrics to future research studies represents a critical development need.

Additionally, injury mechanisms, age categories, mortality endpoints, and occupation were not standardised. This lack of uniformity limits the ability to make comparisons between studies and limits the use of this data in the planning of provincial and national public health initiatives and in assessing trauma system performance over time. Similarly, the ability to draw international comparisons of system performance is limited.

The quality of data collected is a limitation of a number of the studies, particularly those using the NISS reporting card, as noted by Zhou et al [26] and Li et al [44]. Both Zhou et al. and Li et al. highlighted problems of missed patients, errors, blanks or illegible items in the Reporting Cards and data entry errors. Similarly, in a pilot study for the NISS designed to examine quality control issues in the ED-based surveillance system, Xie et al [45] reported that 291 out of 1286 registered patients were 'missed' (or inadvertently excluded) by the surveillance system, and of 941 Reporting Cards 5.2% were found to contain errors or blanks in the cards and an additional 19% were found to have data entry errors. The studies reported in this *Review *provide no data to highlight the extent of these concerns.

The scale of the research conducted in these research studies is indicative of the burden of injury facing China's sizeable population. Despite the limitations in the data reported in these studies, the detail relating to injury mechanism age can provide public health specialists with sufficiently high level information to develop targeted intervention campaigns. The ability to undertake planning and quality assurance processes would however be significantly enhanced by the adoption of uniform standards in the collection and reporting of clinical data, such as the Utstein template [13,14] and the ACS *Guidelines *[9], a need clearly highlighted by this *Review*.

### Findings relating to mechanism of injury and patient characteristics

In the setting of provincial, national and global public health priorities, the value of comparable data across jurisdictions cannot be understated. The studies reviewed here highlight the importance of transport, falls, and industrial accidents as the most common causes of injury (Table 7). However, assaults and poisoning feature consistently in these studies. Common to all studies was the predominance of males by a ratio of 2:1. Despite little overlap in age groups between the studies, young adults consistently represented a high proportion of presentations. Mortality rates ranged from 0.5% to 8% where reported.

Table 7 provides for purposes of comparison the *WHO Global Burden of Disease (GBD) 2004 *incident estimates for injuries 'severe enough to require medical attention'[46]. The GBD uses ICD-10 to categories external cause of injury and while direct comparison is imperfect, some observations can be made. The rate of poisonings among the ED patients in China appears high, ranging from 4.7% to 8.6% where recorded; in contrast the GBD estimates range from 1.6% to 2.5% in the four regions shown. Within the China series, machine-related injuries, cutting and piercing and 'blunt' injuries were prominent among the leading causes of injury. In both the China series and the GBD, transport and falls were leading causes though the order differs. Interestingly, among the 13 Chinese papers reviewed those that included suicide did not code poisons and vice versa, potentially highlighting a significant issue in coding practices.

Table 7 also shows for purposes of comparison injury causes in Australia [47], the US [48] and Europe [49]. The Australian (2005-06) data is based on administrative hospital admission datasets that use ICD-10 and codes age in five year increments; the gender ratio was 1.5:1 (male to female). The US data relates to the National Trauma Databank of 712 hospitals and includes the years 2002 to 2006; the male to female ratio was 1.87:1, and notably of the 1,485,098 persons, poisonings and drowning accounted for 0.1% of patients each [48]. The US NTB uses ICD-9-CM and also ISS for all patients irrespective of injury severity. The European Union data (EU-27) relates to fatalities and hospitalisations for the period 2005 - 2007; the mortality data is based on all member states while the hospital admissions data (which is location specific) is assumed to be representative of all EU states. The data is coded is based on the EU Injury Database and information collected by agencies such as EuroStat, and is coded using ICD-10.

It is notable that comparisons based on mechanism using the US, Australian and EU data with the Chinese studies is relatively straightforward. Machine-related injuries, cutting and piercing and poisoning appear more prominent in the studies in China, although road traffic injuries are either the leading or second leading cause of injury across the four jurisdictions. In contrast, fall-related injuries have a lower prominence in the Chinese studies than in the US, Australia and EU regions.

The comparison presented in Table 7 demonstrates that while some comparisons can be made they are imperfect. It is also the case that within the studies in China in this *Review*, the transport/traffic causes cannot be disaggregated into more specific mechanisms of driver, pedestrian etc... while no detail is provided on what constitutes 'blunt' trauma. This provides further weight of evidence that the adoption of internationally recognised data collection and reporting standards in the conduct of injury surveillance research is required.

### Future options for ED injury surveillance research and quality assurance processes - the role of the National Injury Surveillance System and the development of Trauma Registry Systems

In the '25' hospitals study, Chen et al [23] conclude that '*to develop a surveillance post on injuries in the Emergency Departments of general hospitals are not only necessary, urgent, but feasible*.'(pp 209 and 213). Xu et al [27] make a similar point noting that surveillance systems for the basis of injury control strategies, pointing to occupational injury and transport safety as key prevention areas. Statements such as these are indicative of the increasing recognition within China of the need for the establishment of a minimum dataset for the surveillance of injury and the monitoring of trauma outcomes as a means of guiding quality improvement processes and for setting evidence-based health policy. It is also important to recognise that the development of health systems is evolutionary in nature and is a process that is contingent upon the application of resources and the availability of expertise. Undoubtedly the implementation of population-based systems and trauma registry systems is a part of this evolutionary process, the results of which are then utilised to further refine health policy and patient care.

In this context the studies conducted to date and examined by this *Review *could be viewed as precursors of injury surveillance and/or comprehensive trauma registry systems in China. These studies demonstrate both the operational feasibility of these systems and their value as a means of informing public health policy and practice. It is worth noting that the establishment of trauma registry systems is a relatively recent phenomenon globally; for example, the trauma registry system that captures major trauma in Victoria, Australia, was established only a decade ago in 2001 [43]. While China has developed into a leading economic power, this has also occurred only recently [6,50]. While a number of barriers could be suggested for reasons as to why a trauma registry has yet to be established in China - such as language and limited opportunities for training in locations that have established registry systems, it must also be recognised that there is a need to demonstrate the value of such systems which then enables, or 'unlocks' the financial resources required for their initial establishment and on-going operation. This latter point is a particularly important consideration in the context of competing development needs, which remains a feature of China at this point in time - and this is equally applicable in other low and middle income countries.

The development of the *NISS *[36] introduced in 2005 goes some way in addressing the need for a national injury surveillance and registry system. Notably, four of the studies reviewed here used the NISS Reporting Card as the basis for data collection. That the NISS commenced in a limited number of hospitals supports the contention that the development of population-based health data systems is progressive. The NISS now collects information on injuries from 129 hospital emergency departments from 43 counties (20 urban centres, 23 rural centres). Information collected on the Reporting Card includes simple demographics (age, occupation), injury cause information such as time and place of occurrence, causes, intention and activity when injured, as well as time of admission. The Reporting Card also collects information on severity, outcome, clinical diagnosis, and nature and site of injury although internationally recognised scoring systems such as the ICD, ISS, RTS, and TRISS are not currently used. The inclusion of these clinical indicators and severity indices would increase NISS' value immensely, however it is recognized that the necessary training for the use of these indicators is likely to be costly until a point where a collective of local 'train-the-trainers' is established.

It is clear then that the limited use of internationally accepted trauma score metrics combined with the lack of uniformity in data collection and reporting limits the ability of hospitals and relevant health departments to assess the functioning of the trauma system.

This *Review *further highlights the pressing need for the establishment of trauma registry systems to address this gap. While population level public health surveillance systems play a role in determining national health priorities, trauma registries represent a fundamental pillar of any well functioning trauma system by enabling the assessment of individual hospital performance in the treatment of the critically ill and system-wide performance through the examination of recognized Audit Filters [43,51,52]. Such assessments are particularly relevant in developing and expanding trauma systems [53]. Registry data has been utilized to build the evidence base that an integrated and systematic approach to trauma management is associated with a reduction in the incidence of preventable deaths, fewer complications, shorter length of stay and improved functional outcomes [37-42,54-56].

The reviewed studies demonstrate the feasibility of establishing a registry system and as Wang et al [1] note 'China has the financial resources, organisational infrastructure, and public support to rapidly apply lessons from high income countries to achieve international best-practice standards for injury prevention and control...' (p.7). China has both an opportunity and a need to establish a trauma registry system consistent with international standards of core data [9,13-16] with appropriate site specific additions to capture nuances of the health system. Inclusion of these core data points would overcome the limitations in the reporting - and hence comparability, of the studies reviewed here. In addition to performance monitoring and quality control, the ability of trauma registry data to be used to identify injury trends, evaluate public health interventions and provide the basis for capacity building in terms of academic research, educational opportunities and the conduct of clinical trials is significant.

## Conclusions

This *Review *of Chinese-language injury surveillance studies demonstrates that a significant body of hospital-based injury surveillance research has been undertaken in China. These studies were generally impressive in their respective sample sizes and while the majority were prospective collaborative studies, there was a lack of uniformity in reporting key data points. Moreover, none of the studies reported patient data using internationally accepted indices of injury severity. With the incidence of injury in China increasing, commentators have called for the implementation of injury surveillance systems that utilise internationally recognised coding schemes to guide population based public health priority setting. This *Review *supports these calls. While the recently introduced NISS goes some way in addressing the need for a population based injury surveillance system, the lack of uniformity in the reporting of patient information and the limited use of standardised severity indices in the reviewed studies highlights the need for the establishment of hospital-based trauma registry systems. While ambitious - both in terms of the financial and human resources required, the establishment of a trauma registry system and the use of recognised injury severity and outcome metrics are necessary to enable the systematic assessment the functioning of the trauma system, which then provides the means for identifying system improvements and ultimately ensuring the optimal care of the injured patient.

## Competing interests

The authors declare that they have no competing interests.

## Authors' contributions

All authors were involved in study conception, interpreting the results and drafting and revising the paper. MF and JY conducted the search and interpreted the original papers. All authors were involved in the design of the classification system. All authors have read and approve of the final version of the manuscript. MF and JY are the guarantors.

## Pre-publication history

The pre-publication history for this paper can be accessed here:

http://www.biomedcentral.com/1471-227X/11/18/prepub

## Supplementary Material

Additional file 1 **Table S1. Description of categories used to classify identified research papers**. Table with a description of categories used to classify identified research papers, including high level subject domain, primary focus of paper, and subject categorisations. Click here for file

Additional file 2 **Table S2. Number and percent (%) of articles identified by category**. Table with number and percent of articles by classification category. Click here for file
