# Supplementary material for: Injury in China: a systematic review of injury surveillance studies conducted in Chinese hospital emergency departments
Source: BMC Emerg Med. 2011 Oct 26;11:18. doi: 10.1186/1471-227X-11-18 (PMC3219690; doi:10.1186/1471-227X-11-18)
Supplement: Additional file 1 — Table S1. Description of categories used to classify identified research papers. Table with a description of categories used to classify identified research papers, including high level subject domain, primary focus of paper, and subject categorisations. [file 1471-227X-11-18-S1.DOC]

**Table S1. Description of categories used to classify identified research papers**

| **Domain** | **Primary focus** | **Subjects considered** |
| --- | --- | --- |
| Aviation | Air transport | human factors, crashes |
| Burns | Injury of burns | management of wounds, epidemiology, causes |
| Disasters | Natural disasters, floods, earthquakes | health impacts, sanitation |
| Drowning | Drowning | risk factors |
| Hospital-based injury studies | Undertaken within hospitals, patient and injury focussed | surveillance, surgical or medical management, epidemiology and management of injury subtypes; retrospective prediction/prognostic models |
| Medical | Medical disease | basic sciences, behaviour, cardiovascular, genetics, management, neurological, respiratory, traditional Chinese medicine (TCM) |
| Military medicine | Military personnel | wound management, injury patterns, disease spread, history |
| Mortality | Mortality | determinants, notification systems |
| Traffic crashes | Traffic crashes | behavioural factors, hospital-based epidemiological studies, retrospective mortality studies, reviews |
| Non-China study | Not conducted in China | Chinese immigrant health, not in China |
| Occupational Health Safety (OHS) | Workplace, industrial | occupational injuries and management, outcomes, industry specific |
| Other | Other | not classified elsewhere |
| Poisoning | Poisoning | exposure, industrial, benzene, arsenic, radiation |
| Prison population, health | Prisoner-based | traumatic stress |
| Quality of Life / behaviour | Psychology studies | post-injury, validation of quality of life scales |
| Severe Acute Respiratory Syndrome (SARS) | SARS | pathology, system response, commentary |
| Suicide | Rates, correlates | predictors, mortality and hospital studies |
| Systems | Public health; system-based considerations | disaster planning, disease surveillance, system-wide health care, data system designs, injury surveillance (schools, community, mortality), quality, structure, training |
| Violence | Assault, domestic | physical abuse, prevalence |
