# Supplementary material for: Injury in China: a systematic review of injury surveillance studies conducted in Chinese hospital emergency departments
Source: BMC Emerg Med. 2011 Oct 26;11:18. doi: 10.1186/1471-227X-11-18 (PMC3219690; doi:10.1186/1471-227X-11-18)
Supplement: Additional file 2 — Table S2. Number and percent (%) of articles identified by category. Table with number and percent of articles by classification category. [file 1471-227X-11-18-S2.DOC]

**Table S2.** Number and percent (%) of articles identified by category

| **Article category** | **Freq.** | **%** |
| --- | --- | --- |
| Traffic, motor vehicle | 73 | 27.2 |
| **Hospital-based injury studies** | **65** | **24.3** |
| Systems | 62 | 23.1 |
| Medical | 27 | 10.1 |
| Occupational Health | 5 | 1.9 |
| Disasters | 8 | 3.0 |
| Burns | 4 | 1.5 |
| Poisoning | 3 | 1.1 |
| Mortality | 7 | 2.6 |
| Suicide | 1 | 0.4 |
| Severe Acute Respiratory Syndrome (SARS) | 5 | 1.9 |
| Military medicine | 2 | 0.7 |
| Quality of Life / behaviour | 1 | 0.4 |
| Aviation | 4 | 1.5 |
| Other | 1 | 0.4 |
| Violence | 0 | 0.0 |
| Drowning | 0 | 0.0 |
| Non-China study | 0 | 0.0 |
| Prison population, health | 0 | 0.0 |
| Total | 268 | 100 |
